# The NRF2 Activation and Antioxidative Response Are Not Impaired Overall during Hyperoxia-Induced Lung Epithelial Cell Death

**DOI:** 10.1155/2013/798401

**Published:** 2013-04-28

**Authors:** Haranatha R. Potteti, Narsa M. Reddy, Tom K. Hei, Dhananjaya V. Kalvakolanu, Sekhar P. Reddy

**Affiliations:** ^1^Department of Pediatrics, College of Medicine, University of Illinois at Chicago, Chicago, IL 60612, USA; ^2^Mailman School of Public Health, Columbia University, New York, NY 10032, USA; ^3^Department of Microbiology and Immunology and Greenebaum Cancer Center, University of Maryland School of Medicine, Baltimore, MD 21201, USA

## Abstract

Lung epithelial and endothelial cell death caused by pro-oxidant insults is a cardinal feature of acute lung injury/acute respiratory distress syndrome (ALI/ARDS) patients. The NF-E2-related factor 2 (NRF2) activation in response to oxidant exposure is crucial to the induction of several antioxidative and cytoprotective enzymes that mitigate cellular stress. Since prolonged exposure to hyperoxia causes cell death, we hypothesized that chronic hyperoxia impairs NRF2 activation, resulting in cell death. To test this hypothesis, we exposed nonmalignant small airway epithelial cells (AECs) to acute (1–12 h) and chronic (36–48 h) hyperoxia and evaluated cell death, NRF2 nuclear accumulation and target gene expression, and NRF2 recruitment to the endogenous *HMOX1* and *NQO1* promoters. As expected, hyperoxia gradually induced death in AECs, noticeably and significantly by 36 h; ~60% of cells were dead by 48 h. However, we unexpectedly found increased expression levels of NRF2-regulated antioxidative genes and nuclear NRF2 in AECs exposed to chronic hyperoxia as compared to acute hyperoxia. Chromatin Immunoprecipitation (ChIP) assays revealed an increased recruitment of NRF2 to the endogenous *HMOX1* and *NQO1* promoters in AECs exposed to acute or chronic hyperoxia. Thus, our findings demonstrate that NRF2 activation and antioxidant gene expression are functional during hyperoxia-induced lung epithelial cell death and that chronic hyperoxia does not impair NRF2 signaling overall.

## 1. Introduction

The induction of antioxidant gene expression in lung-resident and infiltrated inflammatory cells in response to oxidative stress plays a significant role in pulmonary defense mechanisms [1, 2]. However, disequilibrium between prooxidant load and antioxidant defenses, leading to redox imbalance, could potentially enhance tissue susceptibility to oxidative stress, thereby contributing to the lung pathogenesis of many acute and chronic airway diseases. These diseases include idiopathic pulmonary fibrosis, emphysema, bronchopulmonary dysplasia, acute lung injury (ALI)/acute respiratory distress syndrome (ARDS), and lung cancer [3–6]. Supplemental oxygen (hyperoxia) is used as therapy to treat ALI/ARDS patients. In mice, chronic exposure to hyperoxia results in endothelial and alveolar epithelial cell death accompanied by pulmonary edema and respiratory impairment; these pathologic features are similar to those observed in ALI/ARDS patients [7]. Thus, understanding the mechanisms by which hyperoxia contributes to lung pathogenesis is crucial to limiting the potentially harmful effects of oxygen toxicity in the clinical setting.

We have previously shown that NF-E2-related factor 2 (Nfe2l2, also known as Nrf2), a bZIP transcription factor, is crucial for the induction of several antioxidant and cytoprotective genes in response to various pro-oxidant stimuli, including hyperoxia [8, 9]. Nrf2-deficient mice are more susceptible than wild-type mice to inflammatory and hyperpermeability responses to hyperoxic insult; this response has been generally attributed to a diminished or low expression level of several antioxidant enzymes (AOEs), including gene encoding *NQO1*, *HMOX1*, *GCLC*, *GCLM*, and *GPX*s [8, 9], which detoxify reactive oxygen species (ROS) and/or nitrogen (RNS) species. We have also shown that a loss of *Nrf2* impairs the resolution of hyperoxia-induced acute lung injury and inflammation and also exacerbates bacterial infection in adult mice following hyperoxic insult [10]. 

One-day old Nrf2-deficient pups, when exposed to hyperoxia for 72 h, develop greater levels of alveolar simplification (septal growth arrest) at the 4th day [11] and 14th day [12] than do Nrf2-sufficient pups. Nrf2 deficiency enhances cellular stress and susceptibility to oxidant-induced lung epithelial cell death [13], and its overexpression confers cellular protection against hyperoxia in lung epithelial cells [14] as well against proapoptotic stimuli in nonlung epithelial cells [15, 16]. These observations suggest an important role for the Nrf2-driven transcriptional response in mitigating cellular stress induced by prooxidants. 

Since chronic exposure to hyperoxia causes the death of lung epithelial cells, despite the presence of NRF2, we hypothesized that dysfunctional NRF2 signaling may contribute to this cell death. To test this hypothesis, we have now analyzed the nature of NRF2 activation (nuclear accumulation) and recruitment to the antioxidant gene (*HMOX1* and *NQO1*) promoters in human nonmalignant lung small airway epithelial cells during acute and chronic hyperoxia exposure. Here, we report that chronic hyperoxia does not impair NRF2 nuclear accumulation or antioxidant gene expression during the hyperoxia-induced death of lung epithelial cells.

## 2. Materials and Methods

### 2.1. Human Lung Epithelial Cell Culture and Hyperoxia Exposure

The human normal small airway epithelial cell line (hereafter referred to as AECs) was established by the ectopic expression of human telomerase reverse transcriptase (hTERT). Cells were grown in Dulbecco's Modified Eagle Medium with Ham's F12 nutrient mixture (DMEM/F12) in the presence of 10% FBS and antibiotics [17]. Cells were plated in equal number, grown to 70%–80% confluence and then exposed to hyperoxia in complete medium. For generating hyperoxic condition, cells were kept in modular incubator chamber (Billups-Rothenberg, Del Mar, CA) and filled with gas mixture containing 95% O_2_ and 5% CO_2_ and chambers placed in 37°C incubator. The chambers were refilled with the gas mixture every 12 h. As room air control group, cells were placed in the regular cell culture incubator with room air and 5% CO_2_ at 37°C. Culture medium was changed every 24 h during the exposure period.

### 2.2. Gene Expression Analysis

Cells were exposed to either room air (RA) or hyperoxia (95% O_2_ and 5% CO_2_) for the indicated time periods. Total RNA was isolated using Trizol reagent (Gibco-BRL/Life Technologies, Grand Island, NY) and reverse transcribed using the qScript cDNA SuperMix (Quanta BioSciences, Gaithersburg, MD). Target gene expression was assessed by quantitative RT-PCR (qRT-PCR) using TaqMan gene expression assays (Applied Biosystems, Foster City, CA). For Immunoblot analyses, total protein was extracted in lysis buffer consisting of 20 mM Tris (pH 7.5), 150 mM NaCl, 1 mM EDTA, 1 mM EGTA, 1% Triton X-100, 2.5 mM sodium pyrophosphate, 1 mM Na_3_VO_4_, 5 mM *β*-glycerophosphate, and 1 *μ*g/mL leupeptin. Comparable amount of total protein (~40 *μ*g) from each sample was separated on a 10% sodium dodecyl sulfate polyacrylamide gel electrophoresis (SDS-PAGE), and the membranes were probed with antibodies specific for NRF2 (Santa Cruz Biotech, Santa Cruz, CA), HMOX1 (Santa Cruz Biotech, Santa Cruz, CA), NQO1 (Abcam, Cambridge, UK), GCLC (kindly provided by Dr. Terrance Kavanagh, University of Washington, Seattle, WA). *β*-actin (Sigma, St. Louis, MO) antibody was used as the loading control. The blots were developed using an ECL kit (HyGlo, Denville Scientific Inc., Metuchen, NJ).

### 2.3. Cell Viability Assays

Cells in equal number were plated and exposed to hyperoxia as described above. Cell viability was quantified by CellTiter-Glo kit (Promega, Madison, WI) and MTT assay. LDH release was measured by CytoTox 96 NonRadioactive Cytotoxicity assay kit (Promega, Madison, WI). Viability and LDH release was calculated as a percentage of increase or decrease over their respective room air controls.

### 2.4. Chromatin Immunoprecipitation (ChIP) Assays

ChIP assays were performed using the EZ-ChIP assay kit (Millipore, Billerica, MA). Briefly, AECs were exposed to either room air or hyperoxia for indicated time points, cross-linked with formaldehyde, and chromatin fragmentation was carried out as detailed in the kit procedure. Diluted soluble chromatin solution was incubated with rabbit anti-NRF2 (Santa Cruz Biotechnology, Santa Cruz, CA) for 18 h at 4°C with rotation. Nonimmune rabbit IgG was used as a negative control to determine the binding specificity. Following incubation with protein A/G agarose beads, the bound products were washed, and DNA was eluted. DNA was subjected to PCR with primers encompassing the functional antioxidant response elements (AREs) located upstream of transcriptional start site of *HMOX1* (F: 5′-CCCTGCTGAGTAATCCTTTCCCGA-3′ and R: 5′-ATGTCCCGACTCCAGACTCCA-3′) and *NQO1* (F: 5′-GTGGAAGTCGTCCCAAGAGA-3′ and R: 5′-TGTCTCCCCAGGACTCTCTCAG-3′) to determine the binding of NRF2 in ChIP assays.

### 2.5. Transfections and Reporter Gene Analyses

Cells were transfected with the *NQO1* (NADPH: quinone oxidase reductase-1) promoter reporter (luciferase) construct [18] (kindly provided by Jeffrey Johnson, University of Wisconsin). To normalize transfection efficiency between wells, the cells were cotransfected with 5 ng of the Renilla luciferase plasmid pRL-TK (Promega Corp., Madison, WI). At 24 h after transfection, cells were exposed to either room air or hyperoxia, and extracts were assayed for firefly and the Renilla luciferase activities using a dual luciferase kit (Promega Corp., Madison, WI). Firefly luciferase activity was normalized to that of Renilla. 

### 2.6. Statistical Analyses

Data were expressed as the mean ± SD (*n* = 3–9) as indicated in the legends. The significance between the exposures was calculated by one-way or two-way (for cell viability and LDH release) analysis of variance followed by the Bonferroni post hoc tests by GraphPad PRISM 4 Software. A *P* value of ≤0.05 is considered statistically significant. We also performed Student's *t*-test to confirm one-way analysis.

## 3. Results

**Figure 1:**
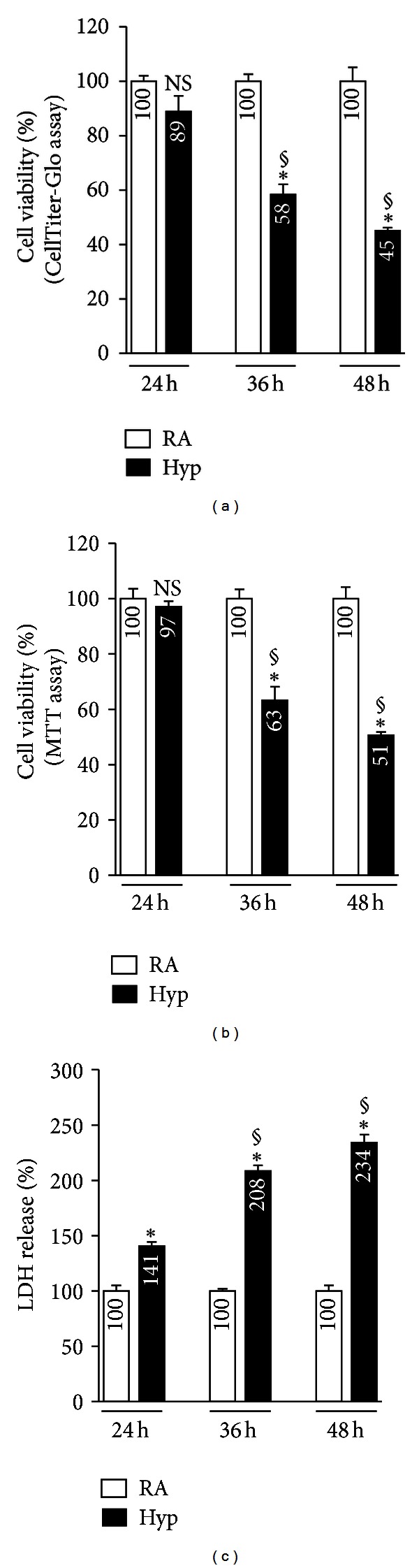
Hyperoxia induced cell death in AECs. Cells were exposed to hyperoxia (hyp) or room air (RA) for up to 48 h, and cell viability was measured at the indicated time points. Cell viability assay was evaluated with CellTiter Glo Reagent (a), MTT reagent (b), and LDH release into the culture medium (c). Results are represented as the percent viability of cells (a and b) or LDH release (c) in comparison to their time matched RA control groups. The graph represents mean ± SD of 4 independent experiments performed in triplicate. *P* values are calculated using two-way ANOVA by Prism software. *P ≤ 0.05, RA versus hyp; §: *P* ≤ 0.05, 24 h hyp versus 36 h hyp or 36 h hyp versus 48 h hyp.

Malignant human lung epithelial cells express higher levels of NRF2 than do nonmalignant lung epithelial cells mainly because of mutations in the inhibitor of NRF2, KEAP1, which is known to facilitate NRF2 degradation under basal conditions [19–21]. To overcome this problem and to determine whether chronic hyperoxia promotes lung epithelial cell death by suppressing the NRF2-mediated transcriptional response, we have utilized a nononcogenic human lung (small airway) epithelial cell line immortalized by telomerase [17]. To assess the effect of hyperoxia on airway epithelial cells (AECs), we subjected the cells to hyperoxia for 24 to 48 h and measured cell viability by using CellTiter-Glo and MTT reagents; LDH release was evaluated by Cytotox. No significant difference in cell viability was observed between room air and hyperoxia during the first 24 h of exposure (Figure 1(a)). However, after 36 h, hyperoxia had produced a significant decline (~42%) in cell viability (as determined by CellTiter-Glo), which fell to ~55% at 48 h. To verify this result, we evaluated cell viability using MTT assay (Figure 1(b)). Hyperoxia caused a significant decline (~37% loss) in cell viability after 36 h, and the loss of viability increased ~49% at 48 h; in contrast, hyperoxia exposure for 24 h had no significant effect on cell viability when compared to room air controls. 

We also analyzed hyperoxia-induced cellular toxicity, as measured by LDH release into the culture medium. A significant increase in LDH release was detected in cells exposed to hyperoxia for 24 h (Figure 1(c)), when compared to their room air-exposed counterparts. However, the LDH released by the cells exposed to hyperoxia for 36 h to 48 h was markedly higher than the amount in the corresponding room air controls (Figure 1(c)). The discordance in results between the cell viability (Figures 1(a) and 1(b)) and LDH measurement (Figure 1(c)) at the 24 h time point may be related to the differences in the sensitivity of the assays.

**Figure 2:**
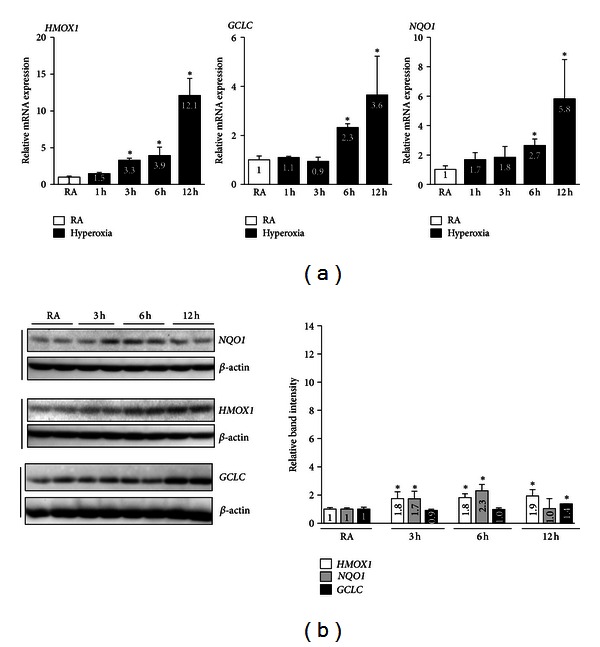
NRF2 target gene expression in AECs exposed to acute hyperoxia. Cells were exposed to either room air or hyperoxia for 1 to 12 h and then harvested at indicated time points for total RNA, and protein extraction. (a) cDNA was prepared from total RNA and the mRNA expression levels of HMOX1, GCLC and NQO1 were quantified using TaqMan assay probes. The values represent the mean ± SD of three independent experiments (*n* = 3). *P < 0.05 RA versus hyperoxia. (b) A comparable amount of whole cell extracts (~40 *μ*g) was separated on SDS-PAGE gel and immunoblotted with anti-HMOX1, anti-NQO1, or anti-GCLC antibodies. *β*-actin was used a as reference. Graph represents the relative band intensities of the HMOX1, NQO1, and GCLC of three independent experiments done in duplicate (*n* = 6). The band intensities were quantified using Image J software and normalized with their respective *β*-actin band intensities. Band intensities of room air exposed samples were considered as one arbitrary unit. *P < 0.05, RA versus hyperoxia.

To determine the impact of chronic hyperoxia on the regulation of the NRF2-dependent antioxidant transcriptional response, we subjected cells to either acute (1–12 h) or chronic (36–48 h) hyperoxia and determined the expression levels of *HMOX1, GCLC,* and *NQO1* by qRT-PCR and immunoblot analysis. We selected these genes because they are upregulated by cellular stress and are putative transcriptional targets of NRF2 and because they are known to play key roles in cellular detoxification processes (reviewed in [22]). As anticipated, acute hyperoxia markedly stimulated *GCLC* (2.3-fold) and *NQO1* (2.7-fold) mRNA expression by as early as 6 h, and the levels remained high up to 12 h (3.6-fold and 5.8-fold increases for *GCLC* and *NQO1,* resp.) (Figure 2(a)). In contrast, a significant increase in *HMOX1* mRNA expression was noticed during the acute phase as early as 3 h (3.3-fold), and its expression remained high (12.1-fold) up to 12 h. To verify that the changes in mRNA expression also occurred at the protein level, we performed western blot analyses using anti-HMOX1, -GCLC, and -NQO1 antibodies on total protein extracts prepared from cells exposed to hyperoxia or normoxia. In agreement with the RT-PCR data, western blot analyses showed an increased expression of these genes (Figure 2(b)). HMOX1 expression was increased by 1.8-, 1.8-, and 1.9-fold at 3 h, 6 h, and 12 h, respectively, when compared to the room air control group. Likewise, NQO1 expression was increased by 1.7-fold and 2.3-fold at 3 h and 6 h, respectively. No increase was found at the 12-h time point. In the case of GCLC, the protein expression was increased by 1.4-fold at the 12-h time point only.

**Figure 3:**
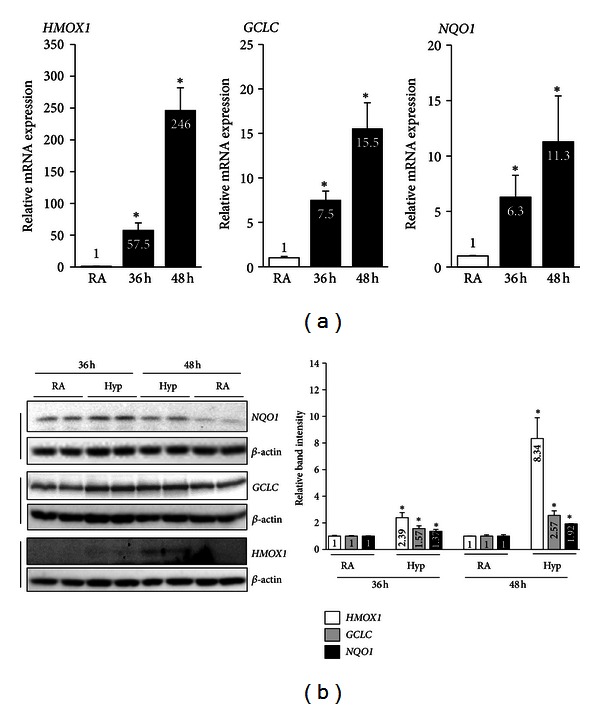
The effects of chronic hyperoxia on NRF2 target gene expression in AECs. Cells were exposed to either room air or hyperoxia for 36 or 48 h and then harvested for total RNA and protein. (a) HMOX1, GCLC, and NQO mRNA expression. The values represent the mean ± SD of four independent experiments (*n* = 6). *P < 0.05, RA versus hyperoxia. (b) Western blot analysis of whole-cell lysates using anti-HMOX1, anti-NQO1, or anti-GCLC antibodies. Membranes were stripped and reprobed with *β*-actin. Graph represents the relative band intensities of HMOX1, NQO, or GCLC, as quantified in Figure 2. Values represent the mean ± SD of four independent experiments (*n* = 4). *P < 0.05, RA versus hyperoxia.

We next analyzed the expression levels of these genes in cells exposed to chronic hyperoxia in order to determine whether their lack of induction by NRF2 could be attributed to cell death. Intriguingly, we found increased levels of *HMOX1* (57.5-fold),* GCLC* (7.5-fold), and *NQO1* (6.3-fold) mRNA expression in cells exposed to chronic hyperoxia (36 h) when compared to those exposed to room air (Figure 3(a)). The induction of *HMOX1* (246-fold), *GCLC* (15.5-fold), and *NQO1* (11.3-fold) mRNA expression remained high up to 48 h. The results of the western blot analyses were correlated with a significantly increased expression of HMOX1 (2.39-fold at 36 h and 8.34-fold at 48 h), GCLC (1.37-fold at 36 h and 1.92-fold at 48 h) and NQO1 (1.57-fold at 36 h and 2.57-fold at 48 h) over the corresponding room air control groups (Figure 3(b)), but the levels of each protein were not reflected by a corresponding mRNA abundance.

**Figure 4:**
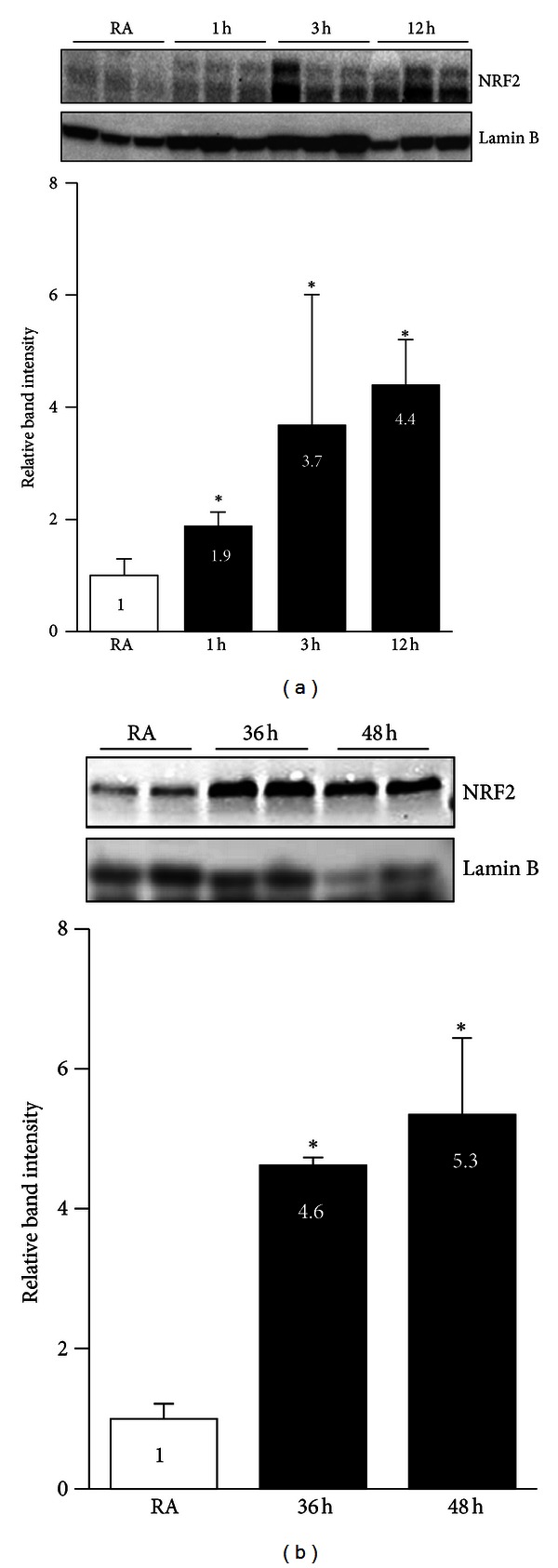
The effects of acute and chronic hyperoxia on nuclear NRF2 levels. Cells were exposed to room air (RA) or hyperoxia for indicated time points and nuclear extracts were prepared. Equal amount of protein (~15 *μ*g) was separated on SDS-PAGE and probed with anti-NRF2 antibody. Membrane was stripped and re-probed with anti-lamin B antibody, as loading control. (a). Nuclear NRF2 levels in cells exposed to hyperoxia for 1 to 12 h. (b) Nuclear NRF2 levels in cells exposed to hyperoxia for 36 h or 48 h. Graph represents the relative NRF2 band intensity of three independent experiments ± SD (*n* = 3). The band intensities of NRF2 were quantified using Image J software and normalized with their respective lamin B band intensities. NRF2 band intensities of room air-exposed samples considered as one arbitrary unit.

In order to examine the effects of acute and chronic hyperoxia on NRF2 nuclear accumulation, we exposed AECs to hyperoxia then prepared nuclear extracts and performed western blotting using an anti-NRF2 antibody. Hyperoxia increased the levels of nuclear NRF2 (Figure 4(a)) as early as 1 h (1.9-fold) after exposure, and the levels remained higher than those of the corresponding room air control group at 3 h (3.7-fold) and up to 12 h (4.4-fold). In response to chronic hyperoxia, NRF2 nuclear accumulation was higher at 36 h (4.6-fold) and 48 h (5.3-fold) than to room air (Figure 4(b)). These results demonstrate that NRF2 accumulates in the nucleus in response to chronic hyperoxia in a manner comparable to that observed in cells exposed to acute hyperoxia.

**Figure 5:**
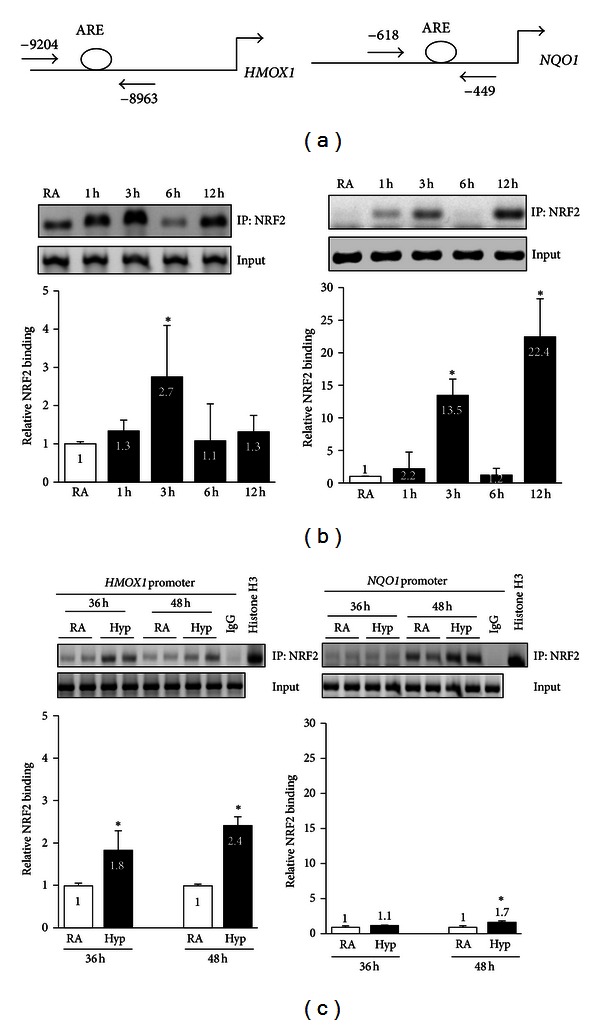
The effects of acute and chronic hyperoxia on the binding of NRF2 to *HMOX1* and *NQO1* promoters. Cells were exposed to room air or hyperoxia for 1 to 12 h or for 36 to 48 h; chromatin was cross-linked and immunoprecipitated with IgG or anti-NRF2 antibodies, and DNA was amplified using gene specific primers. (a) Scheme showing the positions of the ARE sites, forward and reverse primers of *HMOX1* and *NQO1* promoters used in ChIP assays. (b) NRF2 binding to the *HMOX1* and *NQO1* promoters in cells exposed to acute hyperoxia (1–12 h). (c) NRF2 binding to the *HMOX1* and *NQO1* promoters in cells exposed to chronic hyperoxia (36 h or 48 h). PCR products were analyzed on 2% agarose gel. Band intensities were quantified with Image J software. Input DNA was used as a control. Graph represents mean ± SD of five independent experiments (*n* = 5). Fold increase was calculated over their respective room air controls *P < 0.05, RA versus hyperoxia.

To determine whether the increased levels of *HMOX1* and *NQO1* expression are due to NRF2 binding, we performed ChIP assays analyzing the recruitment of NRF2 to endogenous *HMOX1* and *NQO1* promoters in cells exposed to acute and chronic hyperoxia. We used gene-specific primers flanking space the critical AREs in each case (Figure 5(a)). These experiments revealed the recruitment of NRF2 to the *HMOX1* promoter as early as 3 h; the levels returned to baseline at 6 h during acute hyperoxia (Figure 5(b), left panel). NRF2 binding to the *NQO1* promoter was very low or undetectable under conditions of room air, and it rapidly increased as early as 3 h (13.5-fold) but returned to basal levels at 6 h (Figure 5(b), right panel). However, the binding of NRF2 to the *NQO1* promoter rose again at 12 h of hyperoxia (22.4-fold) (Figure 5(b), left panel). An increased enrichment of NRF2 at the *NQO1* promoter at the 12 h time point was also reflected in higher mRNA levels, but western blot analysis revealed no increase in NQO1 protein expression at the 12 h time point, perhaps because of a lag in mRNA translation. 

During chronic hyperoxia, we found that the binding of NRF2 to the *HMOX1* enhancer was significantly higher at 36 h (1.8-fold) and 48 h (2.4-fold) than in cells exposed to room air (Figure 5(c), left panel). Likewise, during chronic hyperoxia, the binding of NRF2 to the *NQO1* promoter, although not higher at the 36 h time point, was significantly higher at 48 h than in cells exposed to room air (Figure 5(c), right panel). However, the level of NRF2 enrichment at the *NQO1* promoter was considerably lower than in cells exposed to acute hyperoxia (Figure 5(b), right panel). 

**Figure 6:**
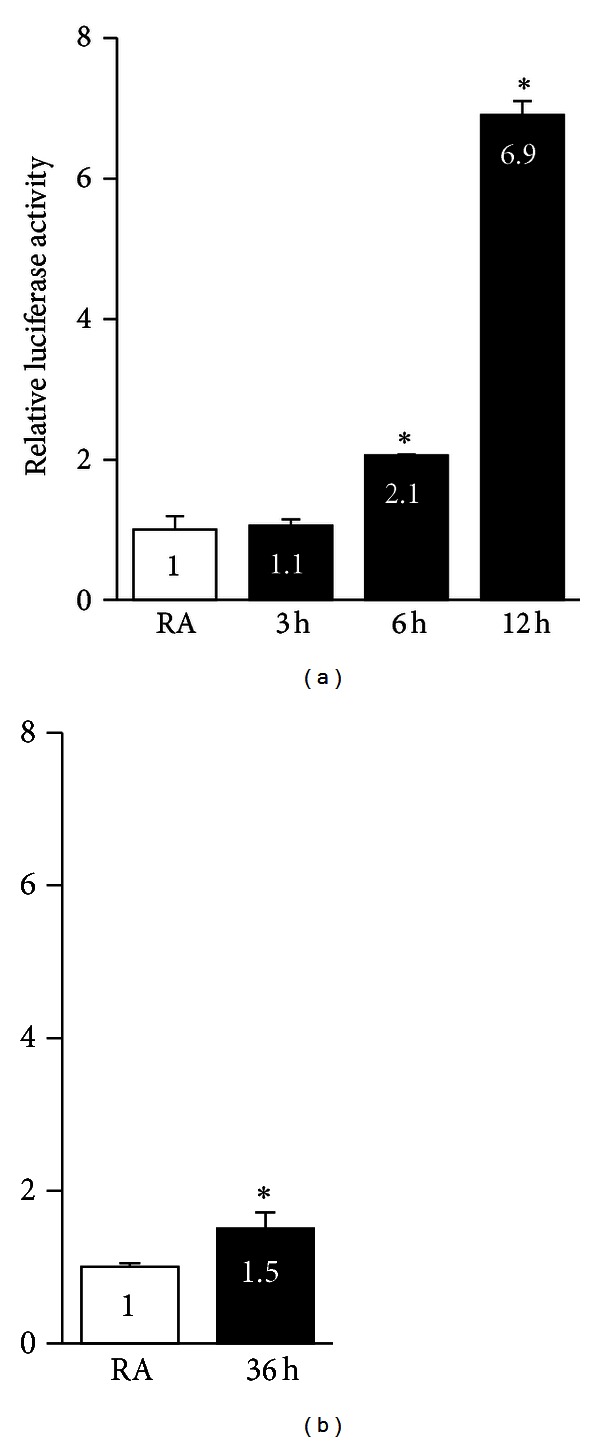
*NQO1* promoter activity in AECs exposed to hyperoxia acutely and chronically. To determine the effects of hyperoxia on *NQO1* promoter activity, luciferase reporter construct bearing *NQO1* promoter (100 ng) and pRL-TK coding for the Renilla luciferase were transiently transfected into AECs, exposed to hyperoxia or room air, and luciferase activity was analyzed using dual luciferase assay kit. The promoter activity of AECs exposed to room air was set as on unit. Values represent the average of 3 independent experiments done in triplicate (*n* = 9).

We next determined the transcriptional activity of the *NQO1* promoter in cells exposed to acute and chronic hyperoxia. Cells were transiently transfected with the *NQO1-Luc* reporter construct and then exposed to hyperoxia. As shown in Figure 6, hyperoxia enhanced *NQO1-Luc* expression by 2.1- and 6.9-fold at 6 h and 12 h of exposure (Figure 6(a)), respectively, when compared to room air controls. We found that the *Luc* activity was also significantly higher (1.5-fold) in cells exposed to chronic hyperoxia for 36 h than in the corresponding room air control group (Figure 6(b)); this result is comparable to the 2.1-fold increase found in cells exposed to hyperoxia for 6 h. However, the magnitude of the promoter inducibility was considerably lower than the 6.9-fold increase observed in cells exposed to hyperoxia for 12 h. 

## 4. Discussion

Exposure to hyperoxia for prolonged periods (chronic exposure) is known to induce oxidative stress, mainly as a result of a redox imbalance caused by excessive accumulation of reactive oxygen species, which initially causes cellular damage and ultimately cell death [23]. NRF2-induced expression of antioxidative and cytoprotective genes is crucial to maintaining redox homeostasis during exposure to toxicants and injurious insults. We have previously shown that mice lacking *Nrf2* are highly susceptible to hyperoxia-induced lung injury and lung epithelial cell death *in vivo* and *in vitro* [8, 14], suggesting that prolonged exposure to hyperoxia promotes lung epithelial cell injury and death by impairing NRF2 activation and subsequently inhibiting its induction of downstream target genes. However, in the present study, we demonstrate that the enhanced expression levels of putative NRF2 target genes in lung epithelial cells exposed to chronic hyperoxia are of a higher magnitude than those observed under acute hyperoxia. Moreover, we observed that the recruitment of nuclear NRF2 to the promoters of antioxidant genes (e.g., *HMOX1* and *NQO1*) in cells exposed to chronic hyperoxia. Thus, it appears that NRF2-mediated gene expression is not globally or largely compromised, but it induced to mount a cytoprotective response to preserve redox homeostasis, thereby helping to maintain cell survival or preventing lung epithelial cell death during chronic hyperoxia. 

NRF2 is mainly localized to the cytoplasm in its native state, and its nuclear accumulation in response to stressful insults is critical for antioxidant gene induction [24, 25]. The nucleocytoplasmic shuttling of NRF2 appears to vary according to the inducer and/or cell type and is regulated by multiple complex mechanisms [24, 25]. For example, various protein kinases, such as PKC [26, 27], ERK1/2 [28], and AKT [29], activated by pro-oxidant exposure, are also known to facilitate and enhance the accumulation of NRF2 in the nucleus. After the signal-induced dissociation from its cytoplasmic inhibitor Keap1, importins facilitate the nuclear translocation of NRF2 [30]. Phosphorylation of NRF2 in the nucleus promotes its nuclear exclusion and subsequent Keap1-mediated degradation [31, 32]. Thus, deregulation of NRF2 nuclear accumulation following chronic stressful stimuli, such as hyperoxia, is generally connected with cellular injury and death. However, our present results indicate that this is not the case. We found higher levels of NRF2 in the nucleus of cells during chronic hyperoxia than in room air-exposed cells, and the levels were comparable to those of cells exposed to acute hyperoxia, suggesting that the various effector pathways that facilitate nuclear accumulation of NRF2 and its DNA binding are functional during conditions of hyperoxia-induced lung epithelial cell death. 

NRF2 alone is insufficient to bind to the ARE. Its heterodimerization with other b-ZIP transcription factors, such as JUN and the small MAF family of proteins, is also required [33–36]. The magnitude and duration of the antioxidant gene expression can be dictated by both cooperative and combinatorial interactions that occur between NRF2 and the MAF/JUN family of proteins. Thus, the impairment of NRF2 binding to the DNA might affect its transactivational activity, despite elevated levels of NRF2 in the nucleus. Because the levels of NRF2-dependent target gene expression are almost higher under chronic hyperoxia than during exposure to acute hyperoxia, it appears that hyperoxia does not alter these interactions or diminish the lung antioxidant capacity, leading to a dysfunctional cellular response. Members of the Jun family of proteins, c-Jun and Jun-D, can dimerize with Nrf2 and upregulate antioxidant gene expression [37, 38]. For example, mouse embryonic fibroblasts lacking *c-Jun* or *Jun-D* demonstrate decreased levels of antioxidant enzymes and enhanced oxidative stress [33–36]. Likewise, c-Myc upregulates the expression of cytoprotective genes in response to stressful stimuli [39]. Thus, it is possible that these proteins act in a cooperative or synergistic manner with NRF2 to potentiate ARE-mediated gene transcription during chronic hyperoxia. 

We have previously reported the binding of Nrf2 to AREs in murine alveolar type-II like epithelial cells following acute hyperoxia [14, 28]. In the present study, ChIP assays revealed the binding of NRF2 to its target gene promoters (*NQO1* and *HMOX1*) in cells exposed to chronic hyperoxia (48 h), suggesting that chronic hyperoxia does not compromise NRF2 binding to antioxidant promoters. Although the magnitude of the NRF2 binding at the *HMOX1* promoter was similar under acute (at 3 h) and chronic (at 36 h and 48 h) hyperoxic conditions (Figure 5), the enrichment of NRF2 at the *NQO1* promoter appeared to be variable and biphasic in response to hyperoxia. There was a 13-fold increase in the binding of NRF2 to the *NQO1* promoter at 3 h, and the binding returned to basal levels at 6 h after hyperoxia. However, the NRF2 binding increased significantly to greater levels at 12 h than at 3 h after hyperoxia. During chronic hyperoxia, a significant increase in NRF2 binding at the *NQO1* promoter was detected at 48 h, but not at 36 h, when compared to room air controls. It is possible that the association of NRF2 with its partners is dynamic or that its negative regulators, such as BACH1 [40] and Fra-1 [41], limit the availability of NRF2 or its partners to form complexes with NRF2, making it differentially bind to the AREs in a gene-promoter context-dependent manner during acute and chronic hyperoxia. The relevance and mechanisms of such differential NRF2 binding to the *NQO1* promoter during hyperoxia-induced cell death warrants a separate investigation. 

It is noteworthy that the lower NRF2 binding at the endogenous *NQO1* promoter was reflected in diminished promoter activation by chronic hyperoxia in our transient transfection assays; however, *NQO1* mRNA expression levels in cells exposed to chronic hyperoxia were nearly comparable to those observed in cells exposed to acute hyperoxia. We assume that the posttranscriptional regulation of *NQO1* explains the discordance between diminished promoter activity and increased mRNA expression of *NQO1* in chronic hyperoxia. It should also be noted that reporter analyses utilize a short fragment of the promoter, unlike the native gene promoter, and this difference may also explain some of the differences. Also, we would like to point out the lack of a direct correlation between mRNA and protein expression. For example, the increased protein abundance of *HMOX1* and other genes analyzed by western blot analysis was ~10-fold less than the corresponding mRNA expression levels in cells exposed to either acute or chronic hyperoxia. It is unclear whether this result reflects error and noise in the real-time PCR and immunoblot analysis and/or variations in protein synthesis and degradation [42]. 

Previously, it has been shown that the exposure to oxidants (such as high doses of H_2_O_2_) leads to decreased NRF2 levels and a consequent suppression of the antioxidant response pathway, culminating in cellular injury and death [43, 44]. However, we found that nuclear NRF2 protein levels in cells exposed to chronic hyperoxia are not reduced when compared to room air-exposed cells or to cells exposed to acute hyperoxia (Figure 4), ruling out such a possibility. The ability of hyperoxia to induce cell death despite the presence of high levels of antioxidant gene expression is somewhat surprising. It is important to note that we have only analyzed the expression of a subset of the NRF2-target genes (*GCLC, HMOX1,* and *NQO1*) (data not shown for *TRX1* and *GCLM*) that are markedly induced during chronic hyperoxia. Previously, by global gene expression profiling and primary lung type alveolar II epithelial cultures from Nrf2-null and wild-type mice, we have shown that Nrf2 regulates the expression of several genes involved in antioxidative and cytoprotective responses as well as cell proliferation and survival [14, 45]. Global mapping ChIP-binding assays and mRNA expression profiling in Keap1-null or Nrf2-null mouse embryonic fibroblasts revealed that Nrf2 binds and regulates the expression of ~500 genes involved in cell proliferation and stress response [46]. It is possible that some of these gene products that dampen the initiation and execution of cell death pathways are not being induced by NRF2 during chronic hyperoxia, despite its nuclear presence; we cannot rule out this possibility. Recently, Taguchi et al. have demonstrated that an increased level of NRF2 accumulation promotes liver damage in autophagy-deficient mice [47]. Whether high levels of nuclear NRF2 have any role in mediating cellular injury during chronic hyperoxic setting remains to be investigated.

In summary, we have demonstrated that both NRF2 nuclear enrichment and upregulation of antioxidative gene expression occur in cells exposed to chronic hyperoxia. ChIP assays revealed that the magnitude of the NRF2 binding at the antioxidant gene promoters is dynamic, variable, and biphasic in response to hyperoxia exposure, suggesting that NRF2-mediated signaling is not globally or largely compromised. Rather, it continues to be functional during chronic hyperoxia that causes the death of lung epithelial cells.
